# Characterization of Collapsin Response Mediator Protein 2 in Colorectal Cancer Progression in Subjects with Diabetic Comorbidity

**DOI:** 10.3390/cells11040727

**Published:** 2022-02-18

**Authors:** Yih-Hsin Chang, Hui-Ju Yang, Huan-Wen Chen, Chiao-Wan Hsiao, Yi-Chen Hsieh, Yu-Wei Chan, Shu-Wen Chang, Wei-Lun Hwang, Wei-Shone Chen, Hou-Hsuan Cheng, Teh-Ying Chou, Fu-Pang Chang, Hsiang-Ling Ho, Fang-Yeh Chu, Yu-Li Lo, Chun-Jung Chen, Hui-Fang Tsai, Ming-Yuh Shiau

**Affiliations:** 1 Department of Biotechnology and Laboratory Science in Medicine, National Yang Ming Chiao Tung University, Taipei 112, Taiwan; cyh@nycu.edu.tw (Y.-H.C.); young19780330@yahoo.com.tw (H.-J.Y.); a94avon6227@gmail.com (H.-W.C.); hcwalicia.ls03@nycu.edu.tw (C.-W.H.); perfect110828@gmail.com (Y.-C.H.); ricky88359@gmail.com (Y.-W.C.); g128525702@gmail.com (S.-W.C.); wlhwang@nycu.edu.tw (W.-L.H.); 2 Department of Medicine, National Yang Ming Chiao Tung University, Taipei 112, Taiwan; wschen@vghtpe.gov.tw (W.-S.C.); hhcheng2@vghtpe.gov.tw (H.-H.C.); 3 Program in Molecular Medicine, National Yang Ming Chiao Tung University and Academia Sinica, Taipei 115, Taiwan; 4 Department of Nursing, College of Nursing, Hungkuang University, Taichung 433, Taiwan; 5 Division of Colon & Rectal Surgery, Department of Surgery, Taipei Veterans General Hospital, Taipei 112, Taiwan; 6 Department of Pathology and Laboratory Medicine, Taipei Veterans General Hospital, Taipei 112, Taiwan; tychou@vghtpe.gov.tw (T.-Y.C.); fpchang@vghtpe.gov.tw (F.-P.C.); hlho5@vghtpe.gov.tw (H.-L.H.); 7 Department of Clinical Pathology, Far Eastern Memorial Hospital, New Taipei City 220, Taiwan; jacpha@mail.femh.org.tw; 8 Graduate School of Biotechnology and Bioengineering, Yuan Ze University, Taoyuan 320, Taiwan; 9 Department of Medical Laboratory Science and Biotechnology, Yuanpei University, Hsinchu 300, Taiwan; 10 School of Medical Laboratory Science and Biotechnology, Taipei Medical University, Taipei 110, Taiwan; 11 Department and Institute of Pharmacology, National Yang Ming Chiao Tung University, Taipei 112, Taiwan; yulilo@nycu.edu.tw; 12 Department of Medical Research, Taichung Veterans General Hospital, Taichung 407, Taiwan; cjchen@vghtc.gov.tw; 13 Department of Medical Laboratory Science and Biotechnology, China Medical University, Taichung 404, Taiwan; 14 Department of Medical Laboratory and Biotechnology, Chung Shan Medical University, Taichung 402, Taiwan; hftsai@csmu.edu.tw; 15 Clinical Laboratory, Chung Shan Medical University Hospital, Taichung 402, Taiwan

**Keywords:** hyperglycemia, CRMP2, colorectal cancer, type 2 diabetes mellitus

## Abstract

Background: Common demographic risk factors are identified in colorectal cancer (CRC) and type 2 diabetes mellitus (DM), nevertheless, the molecular link and mechanism for CRC-DM comorbidity remain elusive. Dysregulated glycogen synthase kinase-3 beta under metabolic imbalance is suggested to accelerate CRC pathogenesis/progression via regulating collpasin response mediator protein-2 (CRMP2). Accordingly, roles of CRMP2 in CRC and CRC-DM patients were investigated for elucidating the molecular convergence of CRC and DM. Methods: CRMP2 profile in tumor tissues from CRC and CRC-DM patients was investigated to explore the link between CRC and DM etiology. Meanwhile, molecular mechanism of glucose to regulate CRMP2 profile and CRC characteristics was examined in vitro and in vivo. Results: CRMP2 was significantly lower in tumor lesions and associated with advanced tumor stage in CRC-DM patients. Physiological hyperglycemia suppressed CRMP2 expression/activity and augmented malignant characteristics of CRC cells. Hyperglycemia promotes actin de-polymerization, cytoskeleton flexibility and cell proliferation/metastasis by downregulating CRMP2 profile and thus contributes to CRC disease progression. Conclusions: This study uncovers molecular evidence to substantiate and elucidate the link between CRC and T2DM, as well as characterizing the roles of CRMP2 in CRC-DM. Accordingly, altered metabolic adaptations are promising targets for anti-diabetic and cancer strategies.

## 1. Introduction

The prevalence of colorectal cancer (CRC) has been globally increasing in the past two decades. CRC is one of the most prevalent cancer types and the cause of cancer mortality worldwide. Approximately 1.5 million new cases are reported each year. According to the statistics of Cancer Registry Annual Report, Health Promotion Administration, Ministry of Health and Welfare, CRC is the most prevalent malignancy and the third cause of cancer mortality in Taiwan. As a matter of fact, Taiwan is the country with the highest CRC incidence of 45.1/100,000, more than 15,000 new cases are diagnosed annually.

Type 2 diabetes mellitus (T2DM) is another disease with climbing prevalence and mortality worldwide. Diabetic patients with long duration or poor disease control are susceptible to multiple complications which lead to severe physiological and economic burdens to the patients, their family, and our society. CRC patients are prone to having diabetic comorbidity [1]. Accumulating evidence shows CRC incidence in diabetics is about twice as high as in the non-diabetic population, approximately 12~20% of diabetic patients are at risk of suffering from CRC [2,3]. Likewise, diabetic patients are statistically more susceptible to developing CRC [4]. In this context, understanding the convergence between CRC and DM comorbidity is crucial for developing effective strategies to tackle the trend of this rapidly increasing CRC and diabetic incidence.

Epidemiological studies identify common demographic risk factors leading to CRC and T2DM, such as aging, lifestyle, and food intake habits. Moreover, T2DM, insulin resistance, and hyperinsulinemia are proved to be independent risk factors for CRC incidence [5,6,7]. Dysregulated glycolytic activity and fatty acid metabolism are implicated in CRC tumorigenesis [8,9,10]. However, the molecular link and mechanism for CRC-DM comorbidity remain elusive even though CRC and T2DM are statistically associated with each other according to the experiences in clinical settings and epidemiological evidence.

The collapsin response mediator protein (CRMP) family is composed of five cytosolic phosphoproteins that are highly expressed in the developing nerve system. CRMP2, the most studied CRMP isoform, plays crucial roles in controlling neuronal outgrowth and polarity [11,12,13]. Altered CRMP2 levels have been observed in several malignant tumors [14]. In particular, CRMP2 is abundantly expressed in 60% of CRC tumor cells and correlated with patients’ clinical manifestations, and therefore, is considered as a potential CRC marker [15]. Once activated by extracellular signals, CRMP2 interacts with several proteins in microtubule-based transport systems to promote microtubule assembly for axon outgrowth [16]. Being phosphorylated at threonine 514 (Thr514) by glycogen synthase kinase-3 beta (GSK-3β) suppresses CRMP2 activity [17]. While the participation of CRMP2 in neuronal development has been extensively studied, its function in tumorigenesis remains to be investigated.

Our most recent study reveals molecular evidence that CRMP2 exerts multiple functions in adipogenesis and lipid deposits through mediating cell proliferation, glucose/lipid metabolism, and cytoskeleton dynamics [18]. The most documented signaling resulted in GSK-3β inactivation being triggered by insulin through PI3K-Akt signaling pathway [19]. This insulin-driven inactivated GSK-3β loses its kinase activity to phosphorylate glycogen synthase, thus plays a critical role in promoting hepatic glycogen synthesis [20]. In addition, GSK-3β modulates microtubule dynamics through regulating CRMP2 activity. Given that mutation of gene encoding adenomatous polyposis coli (APC) is correlated with CRC metastasis through regulating cellular junction and microtubule stability, attachment and migration of CRC cells by binding to β-catenin, tubulin, and GSK-3β [21], it is reasonable to infer that GSK-3β is the junction between insulin signaling and the CRMP2-controlling mechanism, making CRMP2 the link of pathogenesis between CRC and metabolic abnormalities. 

By combining the evidence regarding the fact that CRMP2 is a CRC biomarker [15] and our finding that CRMP2 participates in lipid metabolism and deposits [18], it was logical to hypothesize that CRMP2 is implicated in the etiology of CRC-DM comorbidity through regulating glucose/lipid metabolism, as well as CRC pathogenesis and/or progression. Therefore, this study aimed at investigating CRMP2 expression in tumor tissues from CRC and CRC-DM patients to explore the molecular linkage between CRC and DM etiology. The corresponding molecular mechanism and involvement of metabolic status, glucose levels in particular, to the regulation of CRMP2 profile was examined as well.

## 2. Materials and Methods

### 2.1. Reagents

Antibodies against CRMP2, Thr-514 phosphorylated CRMP2 (pCRMP2), GSK-3β, and pGSK-3β Ser21/9 were purchased from Cell Signaling Technology (Danvers, MA, USA); ECL reagent from Calbiochem (Merck Millipore, Billerica, MA, USA), Trizol Reagent from Thermo Fisher Scientific (Waltham, MA, CA, USA), streptozotocin (STZ) from MilliporeSigma (Burlington, MA, USA), TransIT-X2 reagent form Mirus Bio.

### 2.2. Study Subjects

Decoded residual colon cancer tissues from 61 patients with colorectal cancer (CRC; including 20 females and 41 males, average age 68.2 ± 13.6 years old) and 46 CRC patients with T2DM (CRC-DM; including 17 females and 29 males, average age 73.5 ± 11.5 years old) who received surgery during 2008~2013 at Taipei Veterans General Hospital were used in this study. None of the subjects had radio- or chemotherapy prior to surgery. The demographic data of study subjects including age, gender, body mass index (BMI), biochemical profiles and manifestations (total cholesterol, triglycerides, CRC tumor stage, and CRMP2 expression) are listed in Table 1. The protocols were reviewed and approved by the Institutional Review Board of Taipei Veterans General Hospital (2013-07-006AC), with all the methods carried out in accordance with the approved guidelines.

### 2.3. Immunohistochemical Staining of CRMP2

CRMP2 proteins were immunohistochemically assessed on air-dried 4 μm formalin-fixed, paraffin-embedded sections using NovoLink Polymer Detection System (Leica Microsystems). Briefly, the sections were placed in a microwaveable container, submerged in 10 mM citrate buffer (pH 6.0), wrapped in vented cling film, and incubated for two 5-min periods at a maximum power in a domestic microwave. Following microwaving, the sections were allowed to equilibrate to room temperature in the buffer, and then rinsed in distilled water. CRMP2 antibodies (Cell Signaling) were applied to the sections for 90 min at room temperature. Immunoreactivities were demonstrated according to the manufacturer’s instructions. The sections were counterstained in hematoxylin and semiquantitatively scored by two pathologists independently [22]. Cytosolic immunoreactivities were first evaluated by the percentages of cells showing cytosolic positive staining and staining strength. For the percentages of cells with cytosolic positive staining results, the immunoreactivies were scored as 1+ if 0–1 out of 100 (0–1/100) tumor cells showed positivity; 2+, 3+, 4+, and 5+ when <10%, <1/3, <2/3, and >2/3 of tumor cells with positivity, respectively. For the staining strength, the immunoreactivies were scored as 1, 2, and 3, respectively, with light yellow, yellow, and brown-yellow staining. The CRMP2 expression profile of each study subject was documented by adding the above two scores, with 0~2 recorded as 0^+^, 3~4 as 1^+^, 5~6 as 2^+^, and 7~8 as 3^+^; then, CRMP2 expression was categorized as low (L) with the final scores 0^+^ and 1^+^, and as high (H) with the final scores >2^+^ in Table 2.

### 2.4. Cell Culture

Human colorectal adenocarcinoma SW480 and SW620 cells were maintained in Leibovitz’s L-15 medium (Sigma) supplemented with 10% FBS, penicillin/streptomycin and L-glutamine; HCT15 and HCT15-Snail were cultured in RPMI-1640 medium (Gibco^TM^-, Thermo Fisher Scientific) supplemented with 10% FBS as described [23]. All cells were cultured in a humidified incubator at 37 °C under 5% CO_2_. For glucose experiments, cells were cultured in media with either high (450 mg/dL, HG), medium (200 mg/dL), or physiological low (100 mg/L, LG) glucose.

### 2.5. Animal Experiments

Mouse-colitis-associated colorectal cancer was induced as described [24]. Healthy male C57BL/6 mice were raised in a well-controlled environment (25 ± 2 °C, 40 ± 0% relative humidity, and a 12:12 light dark cycle) with free access to water and food. Mice were randomized into 5 groups: 1] WT control (*n* = 6) with free access to regular water and standard chow diet till the end of the experiment (24-wk old); 2] diet-induced obesity (DIO, *n* = 4), feeding with high-fat diet (HFD) for 16 weeks since 8-wk old; 3] DM mice, inducing type 2 diabetic onset by *i.p.* STZ injection (50 mg/kg) 3 times within the 9th week after DIO mice consumed 8 weeks of HFD (*n* = 4) as described [25]; 4] colitis-associated CRC mice (colitis- CRC, *n* = 5), receiving a single *i.p.* AOM administration (10 mg/kg) at 6-wk old, followed by feeding with drinking water containing 2% DSS for 4 consecutive days at 7-wk and 10-wk old; 5] CRC-DM (*n* = 5), by inducing diabetic and CRC onset in DIO mice by combining HFD, STZ, AOM, and DSS treatments as the time described above. At the end of the experimental procedure (24-wk old), sera samples from all mice were collected for analyzing blood biochemical parameters, then the mice were sacrificed and intestinal tissue samples (from cecum to the anal verge) collected for further analysis. All samples were stored at −80 °C until analysis. For IL-4 treatment, the experiments were conducted as described [26]. Serum glycerol and FFAs were measured after overnight fast using Free Glycerol Determination Kit and Free Fatty Acid Quantification Colorimetric/Fluorometric Kit (K612; BioVision, Mountain View, CA, USA), respectively. Animal protocols were reviewed and approved by the Institutional Animal Care and Use Committee, Hungkuang University (HK-P-10919).

### 2.6. Western Blot Analysis

Cell lysates were prepared in RIPA buffer containing protease inhibitors as described [25]. In total, 60 μg of cell extracts were subjected to SDS-PAGE, transferred to PVDF membrane, and blotted with specific primary antibodies. For detection, membranes were incubated with secondary antibodies for 1 h, and results were visualized with ECL regent (ECL kit, Amersham Biosciences, Piscataway, NJ, USA), then quantified by Labscan software.

### 2.7. RNA Extraction and RT-PCR

Total RNA was isolated using Trizol reagent. Briefly, cDNA was synthesized using 5 μg RNA, 200 pmol oligo dT primer and 5× MMLV RT. Then, 3 μL of first-strand cDNA was amplified using target sequence-specific PCR primer sets (CRMP2: 5′-CGCCGCTAGAGGTGAAATTCT-3′ and 5′-CATTCTTGGCAAATGCTTTCG-3′; GAPDH: 5′-ACCACAGTCCATGCCATCAC-3′ and 5′-TCCACCACCCTGTTGCTGTA-3′). All RT-PCR reactions were carried out with BIO-RAD PCR iCYCLER instrument. Amplified products were identified by electrophoresis on 2% agarose gel. The quantitative results were presented as the mean of three independent experiments.

### 2.8. Proliferation and Wound Healing Assay

For proliferation assay, cells were seeded onto 96-well cell culture plates at a density of 1 × 10^4^ cells/well. Then, 20 μL of Promega™ CellTiter 96™ AQueous One Solution Cell Proliferation Assay was added to each well for a 2 h incubation at 37 °C according to the manufacturer’s instructions. Absorbance at 490 nm was read with an automated plate reader. For wound healing assay, 3 × 10^5^ cells were seeded onto each well in 6-well plates. Once the cells reached 80% confluence, wounds were generated by scratching the cells with a 200 µL pipette tip. Then, cells were washed with serum free medium. Images of the cells were captured at the time indicated, with scratching area calculated using ImageJ as [1−(scratching area at the time indicated)/scratching area at the 0 h].

### 2.9. CRMP2 Knockdown and Overexpression

Cells were transfected with either 20 μM non-specific scramble small interfering RNA (scramble siRNA, 5′-UAAGGCUAUGAAGAGAUACUU-3′) or CRMP2-specific siRNA (5′- GGAUCACGGGGUAAAUUCCUU-3′) by TransIT-X2 reagent according to the manufacturer’s instruction. For CRMP2 overexpression, cells were transfected with 1 *u*g of pTARGET^TM^-CRMP2-Myc using PolyJet^TM^ reagent (SignaGen Laboratories, Rockville, MD, USA) as described [18].

### 2.10. Statistical Analysis

The differences in CRMP2 expression among gender, tumor stage, grade, and TMN value were calculated by χ^2^ test. Logistic regression analysis was used to identify important variables for CRMP2 expression. The difference between groups was evaluated by χ^2^ test or Fisher’s exact test for categorical data (sex, histological subtype, T2DM). Level of statistical significance was defined as *p* < 0.05.

## 3. Results

### 3.1. Significant Association between CRMP2 and Diabetic Comorbidity among CRC Patients

**Figure 1:**
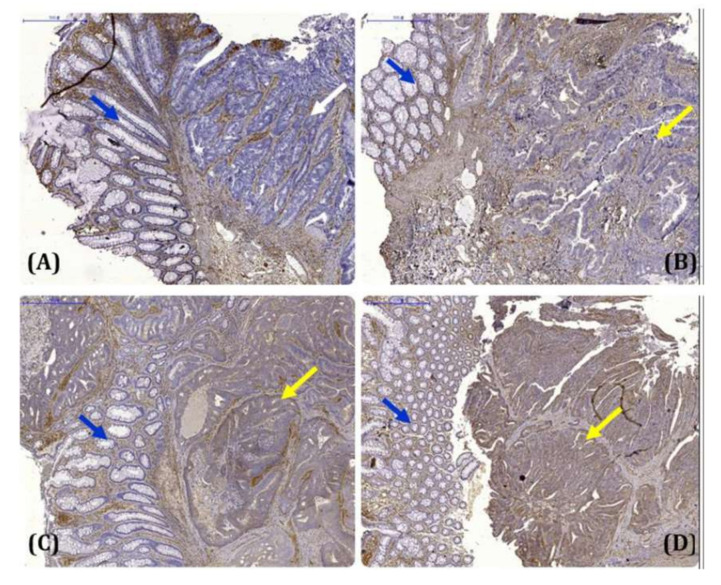
Representative results of CRMP2 immunohistochemical staining in human colorectal cancer tissues. (**A**) Negative immunostaining of CRMP2 in both tumor cells (white arrow) and the adjacent non-tumor cells (blue arrow) in the colorectal cancer tissue section; (**B**) weak immunostaining of CRMP2 in tumor cells (yellow arrow) and negative staining in the adjacent non-tumor cells (blue arrow); (**C**,**D**) strong immunostaining of CRMP2 in tumor cells (yellow arrow) and negative staining in the adjacent non-tumor cells (blue arrow).

CRMP2 expression was successfully examined in tissue sections from all study subjects. Differential CRMP2 staining intensities were detected, whereas the adjacent non-cancerous cells showed faint to negative CRMP2 staining (Figure 1). Notably, a significant difference in CRMP2 levels in tumor loci between CRC and CRC-DM subjects was identified (*p* < 0.0001, Table 1): 17 (27.9%) and 44 (72.1%) CRC subjects had low and high CRMP2 staining intensity, respectively, while the corresponding prevalence of low and high CRMP2 levels in CRC-DM patients was 36 (78.3%) and 10 (21.7%). When the study subjects were stratified by CRMP2 staining score, frequency of higher CRMP2 expression was significantly lower in CRC-DM patients (Table 2, 2^+^: *p* = 0.002 with OR 0.027~0.426, and 3^+^: *p* < 0.0001 with OR 0.006~0.222). These data reveal that CRC-DM patients have lower CRMP2 levels in their tumor lesions. Except for CRMP2 levels (*p* < 0.0001) and circulatory triglycerides (*p* = 0.007), no significant association concerning other demographic information or clinical manifestations between the study subjects was observed (Table 1).

**Figure 2:**
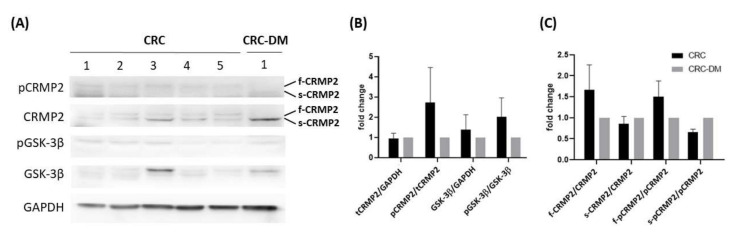
CRMP2 expression profile in tumor loci from CRC and CRC-DM subjects. (**A**) Western blotting results showing CRMP2/pCRMP2 and GKS-3**β**/*p*-GSK3β levels. (**B,C**) Quantification of (**A**); CRC (*n* = 5) and CRC-DM (*n* = 1).

Meanwhile, CRMP2 and pCRMP2 of the tumor loci from CRC (*n* = 5) and CRC-DM (*n* = 1, diabetic duration 22 yrs before being diagnosed as CRC) subjects were examined by Western blot analysis although only one CRC-DM sample was available. Total CRMP2 (tCRMP2, composed of full-length CRMP2 (f-CRMP2, ~62 kDa) and small CRMP2 (s-CRMP2, ~58 kDa); [18]) was rather consistent between CRC and CRC-DM, notably, s-CRMP2 was the major form detected in CRC-DM. Phosphorylated tCRMP2 (pCRMP2) and GSK-3β (pGSK-3β) were decreased in CRC-DM patients, however, phosphorylated f-CRMP2 (f-pCRMP2) and s-CRMP2 (s-pCRMP2) was the major pCRMP2 identified, respectively, in CRC and CRC-DM subjects (Figure 2). The results suggest that f-CRMP2 and s-CRMP2 are the predominant isoforms expressed in CRC and CRC-DM subjects, respectively, and thus, f-pCRMP2 and s-pCRMP2 is the corresponding major pCRMP2 detected in their tumor lesions.

### 3.2. Effects of Glucose on CRMP2 Profiles and Malignant Traits of Colorectal Cancer Cells

The evidence that diabetic status is associated with CRMP2 expression pattern in CRC patients suggested hyperglycemia mediated CRMP2 expression profile. Therefore, it was intriguing to explore the underlying molecular mechanism.

**Figure 3:**
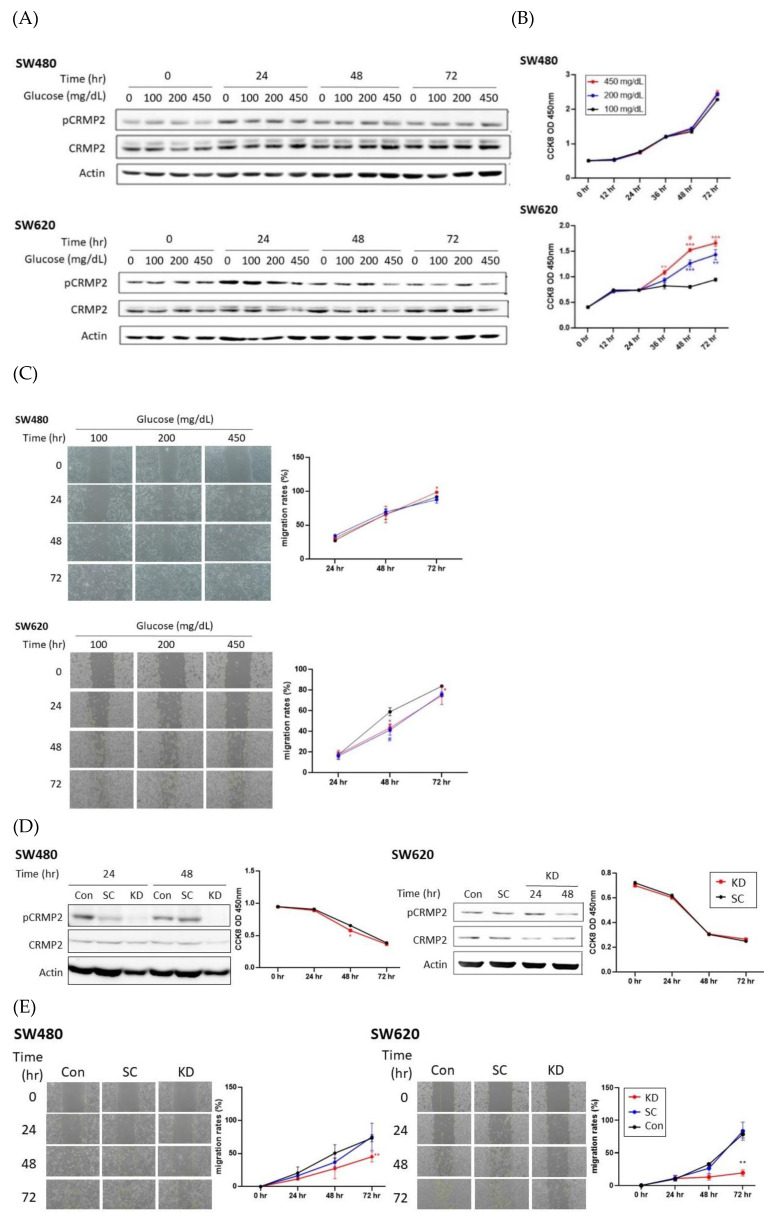
Effect of glucose and CRMP2 silencing on SW480 and SW620 cells. Results of CRMP2/pCRMP2 expression profile (**A**), cell proliferation (**B**), and wound healing ability (**C**) of cells under environments containing differential glucose concentrations for the time periods as indicated. * *p* < 0.05, ** *p* < 0.01, and *** *p* < 0.001 vs. 100 mg/dL; ^#^ *p* < 0.05, vs. 200 mg/dL. Effects of CRMP2 silencing on cellular proliferation (**D**) and wound healing ability (**E**). Data were presented as mean ± SEM (*n* = 3), and statistically analyzed by two-way ANOVA. * *p* < 0.05 and ** *p* < 0.01, vs. scramble.

CRMP2 expression and activity were examined in pairwise in situ SW480 (Dukes’ type B) and metastatic SW620 (Dukes’ type C) CRC cell lines to elucidate the roles of CRMP2 in CRC cells with distinctive malignant potential under differential glucose-containing environments. While tCRMP2 was rather consistent in SW480 cells in the presence of glucose treatment, s-CRMP2 was the predominant isoform (Figure 3A). For metastatic SW620, s-CRMP2 was also the major isoform detected. Notably, both CRMP2 and pCRMP2 tended to reduce accompanied with cell growth and glucose treatment, particularly under hyperglycemia exposure (Figure 3A). While glucose did not greatly affect growth and migration ability of SW480 except for long-term treatment (72 h), proliferation rates were significantly increased but migration ability was only slightly mediated by glucose in SW620 (Figure 3B,C).

The above data echo our previous study [18] that s-CRMP2 is mainly coupled with cell growth and would be downregulated when cells are approaching contact-inhibition. The results imply that glucose is necessary to provide massive energy demands for compensating the downregulated s-CRMP2 in SW620. On the contrary, glucose causes minimal effects on SW480 proliferation due to the consistent s-CRMP2 levels.

Effects of CRMP2 knockdown (KD) were subsequently investigated. Phosphorylated CRMP2 were significantly reduced in SW480 and SW620 cells transfected with CRMP2 siRNA while CRMP2 proteins were relatively less affected (Figure 3D). Cell proliferation was not prominently altered by CRMP2 silencing (Figure 3D) while the wound-healing ability was significantly inhibited by CRMP2 KD (Figure 3E). The above data support our inference that s-CRMP2 is associated with cell proliferation while pCRMP2 is coupled with cell mobility. Therefore, glucose tends to mediate cell proliferation rate in metastatic SW620 cells by providing ambient nutrients to meet the energy demands supporting rapid cell growth. The downregulated inactive pCRMP2 is more likely to be associated with the CRC cell migration trait which echoes the findings in our previous study that morphological flexibility and mobility is tightly regulated by pCRMP2 [18].

**Figure 4:**
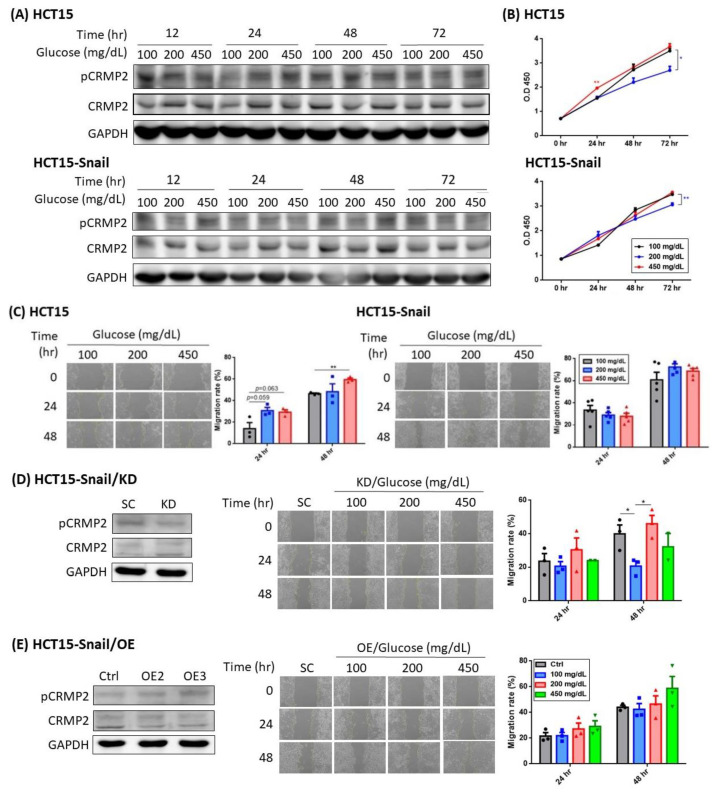
Effect of glucose and CRMP2 on HCT15 and HCT15-Snail cells. Results of CRMP2/pCRMP2 expression profile (**A**), cell proliferation (**B**), and wound healing ability (**C**) of cells under environments containing differential glucose concentrations for the time periods as indicated. * *p* < 0.05 and ** *p* < 0.01, vs. 100 mg/dL. Effects of CRMP2 knockdown (KD) and overexpression (OE) on CRMP2 expression profile (**D**) and wound healing ability (**E**) of HCT15-Snail cells. Data were presented as mean ± SEM (*n* = 3), and statistically analyzed by two-way ANOVA. * *p* < 0.05.

CRMP2 expression pattern was further analyzed in another CRC cell line HCT15 (Dukes’ type C) and its Snail-transfectant designated HCT15-Snail. Epithelial–mesenchymal transition (EMT) activator Snail promotes CRC progression, contributes to treatment failure and poor prognosis [23], therefore, Snail is a potential anti-tumor target as it forms a crucial link between metastasis and stem cell properties in CRC. Snail overexpression (OE) in HCT15 leads to upregulation of numerous downstream genes and thus the manifestations of dedifferentiation traits and most cancer stem cell activities [23]. Both HCT15 and HCT15-Snail exhibited high levels of CRMP2 and pCRMP2, which remained rather consistent under glucose treatment (Figure 4A). Intriguingly, while s-CRMP2 was the predominant isoform detected, levels of f-pCRMP2 and s-pCRMP2 were mostly equivalent. Glucose did not prominently affect cell growth probably due to the abundant s-CRMP2 amounts in both cells supporting cell growth (Figure 4B), whereas higher glucose promoted migration ability of HCT15 cells rather than the de-differentiated HCT15-Snail (Figure 4C).

The effects of altered CRMP2 expression on migration ability were examined next. Unfortunately, migration ability could not be successfully analyzed in CRMP2-OE HCT15 as the transfects would be torn off as a whole piece in the wound-healing assay due to the unexpectedly tight adherence, implying the capacity of CRMP2 to strengthen microtubule stability. Similar to the observations in SW cells, pCRMP2 contributed to cell motility traits (Figure 4D): a transient but significantly reduced wound-healing ability of CRMP2-KD HCT15-Snail under euglycemic environment at 48 h was identified, while the decreased motility was compensated by hyperglycemia (200 and 450 mg/dL). No significant alterations regarding the mobility in the CRMP2-OE counterparts were observed although a trend of glucose-level coupled increasing migration ability was identified (Figure 4E). The data support the observations in SW cells that pCRMP2 is very likely associated with cell migration ability. In addition, the transiently reduced migration ability of CRMP2-KD cells by hyperglycemia implies that glucose concentration mediates morphological flexibility and mobility of CRC cells [18].

### 3.3. Regulation of In Vivo CRMP2 Expression Profile by Diabetic and Colorectal Comorbidity

**Figure 5:**
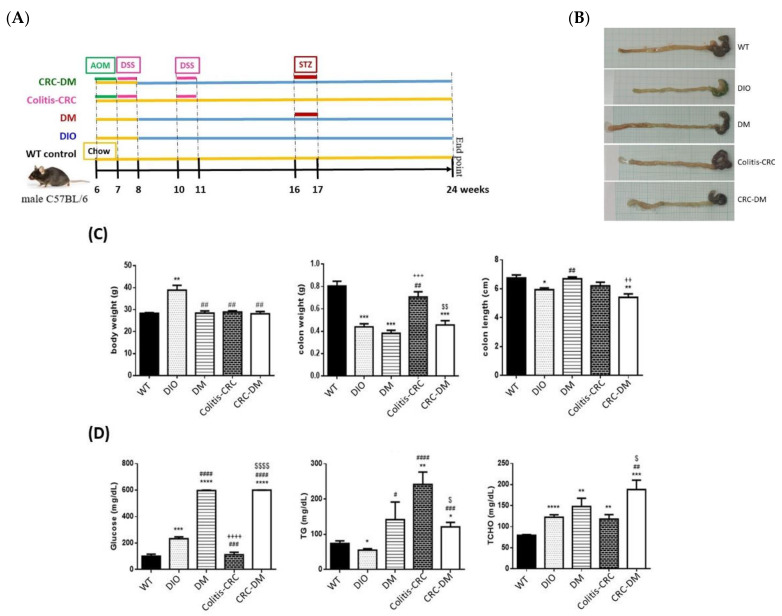
Pathophysiological features of CRC and T2DM mice disease models. (**A**) Schematic diagram showing the experimental protocol for mice with standard diet (WT, *n* = 6), high-diet induced obesity (DIO, *n* = 4), T2DM onset resulted from the HFD-feeding and streptozotocin (STZ) administration (DM, *n* = 4), AOM/DSS-induced colitis associated colorectal cancer (colitis-CRC, *n* = 5), and CRC-DM comorbidity (*n* = 5). (**B,C**) Body weights, colon lengths and weights, (**D**) serum glucose, triglyceride (TG), and total cholesterol (TCHO) were respectively measured. Data were presented as the mean ± SEM, and statistically analyzed by two-tailed unpaired Student’s t-test. * *p* < 0.05, ** *p* < 0.01, *** *p* < 0.001, **** *p* < 0.0001 vs. WT; ^#^ *p* < 0.05, ^##^ *p* < 0.01, ^###^ *p* < 0.001, ^####^ *p* < 0.0001 vs. DIO; ^++^ *p* < 0.01, ^+++^ *p* < 0.001, ^++++^ *p* < 0.0001 vs. DM; ^$^ *p* < 0.05, ^$$^ *p* < 0.01, ^$$$$^ *p* < 0.0001 vs. colitis-CRC.

To further address the roles of CRMP2 in CRC and DM comorbidity in vivo, CRC-DM disease models were generated by combining DIO with AOM/DSS treatment as described in Section 2.5 [24]. In brief, HFD-feeding DIO, DIO mice with diabetic onset (DM) induced by STZ injection, mice with colitis-CRC induced by receiving AOM and DSS administration (colitis-CRC), and diabetic DIO mice with CRC induced by combining all the above treatments (CRC-DM) were established as illustrated in Figure 5A. At the end of the experimental procedure, blood biochemical parameters were examined and colon tissue samples were collected to probe CRMP2 expression profiles.

Symptomatically, colitis-CRC and CRC-DM mice manifested rectum prolapse and bloody stools. Some nodules were macroscopically observed in the intestines of CRC-DM mice (Figure 5B). Significant differences regarding body weights between WT and DIO (28.3 ± 0.7 vs. 38.9 ± 3.9 g, *p* < 0.01) as well as between DIO and the other three disease models (DM, 28.4 ± 1.7 g, *p* < 0.01; colitis-CRC, 28.9 ± 1.1 g, *p* < 0.01; and CRC-DM, 28.2 ± 2 g, *p* < 0.01) were identified (Figure 5C). Colon weight of WT mice (0.8 ± 0.09 g) was significantly higher than DIO (0.44 ± 0.05 g, *p* < 0.001), DM (0.38 ± 0.04 g, *p* < 0.001) and CRC-DM mice (0.46 ± 0.08 g, *p* < 0.001); while that of colitis-CRC mice (0.71 ± 0.09 g) was significantly higher than DIO and DM models. Significant difference in colon length was also identified among these disease models (Figure 5C; WT, 6.8 ± 0.4 cm; DIO, 5.9 ± 0.2 cm; DM, 6.7 ± 0.2 cm; colitis-CRC 6.2 ± 0.5 cm; and CRC-DM, 5.4 ± 0.5 cm). Fasting glucose of mice fed with HFD (DIO, DM, and CRC-DM) was significantly increased (Figure 5D). In particular, prominent hyperglycemia in DM and CRC-DM mice was induced. Differential metabolic abnormalities reflected by blood triglycerides and total cholesterol were also observed in the disease models. In general, lipid panel data were mostly significantly elevated.

**Figure 6:**
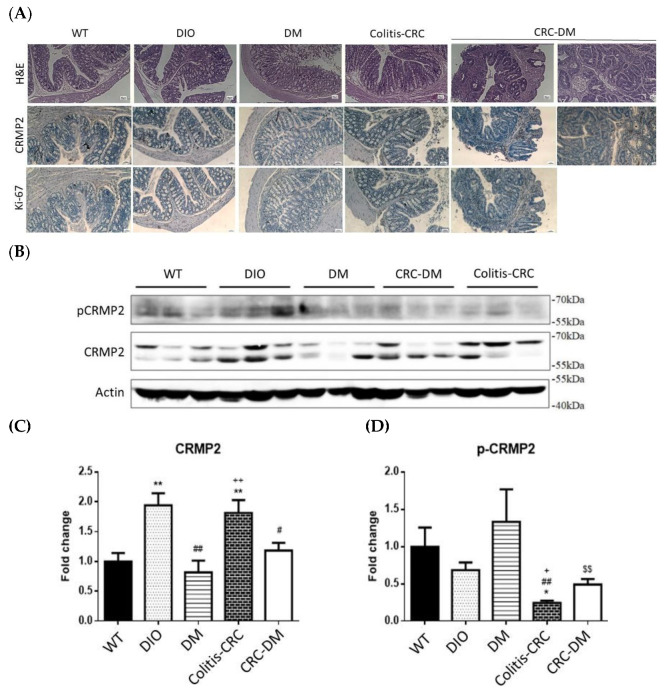
CRMP2 expression and activity in colon tissues from CRC and T2DM mice disease models. (**A**) Representative results showing hematoxylin/eosin staining and CRMP2/Ki-67 immunostaining (CRMP2/Ki-67: brown; nucleus: blue) of colon tissues harvested in the experimental mice models as indicated. Magnification, 100X; scale bar = 50 μm. (**B**) Western blotting results showing CRMP2 profile. (**C**,**D**) Quantification from (**B**). Data were presented as mean ± SEM and statistically analyzed by two-tailed unpaired Student’s t-test. * *p* < 0.05, ** *p* < 0.01 vs. WT; ^##^ *p* < 0.01 vs. DIO; ^+^ *p* < 0.05, ^+ +^ *p*<0.01 vs. DM; ^$$^ *p* < 0.01 vs. colitis-CRC.

Representative results of immunostainings are shown in Figure 6. Histologically, cell morphology was well-preserved in colon tissues harvested from DIO, DM, and colitis-CRC mice. Consistent with the macroscopic observation, pre-cancerous foci with architectural and cytologic atypia were identified in CRC-DM mice. CRMP2 was barely detected in WT and DM mice while DIO and colitis-CRC mice showed faint CRMP2 staining; notably, CRC-DM had stronger CRMP2 staining intensity. Moreover, Ki-67 was weakly detected in WT, DIO, and DM mice but demonstrated stronger staining in colitis-CRC and CRC-DM mice. Loci with stronger Ki-67 staining were superimposed with higher CRMP2 staining areas in CRC-DM mice. Accordingly, CRMP2 levels are promoted among the cells with higher proliferation potential in mice receiving AOM/DSS treatment, and diabetic hyperglycemia is suggested to accelerate CRC pathogenesis in CRC-DM. The finding further supports that CRMP2 is associated with and implicated in CRC tumorigenesis and metastasis.

Colon tissue CRMP2 profile was further investigated by Western blotting (Figure 6B). Total CRMP2 was significantly increased in DIO and colitis-CRC mice which were closely associated with chronic inflammatory status (Figure 6B,C). Intriguingly, s-CRMP2 was the predominant form detected in DIO and CRC-DM mice. The elevated CRMP2 was prominently attenuated by diabetic onset in DM (vs. DIO) and CRC-DM (vs. colitis-CRC) mice, supporting the significantly decreased CRMP2 in CRC patients with diabetic comorbidity. In addition, pCRMP2 of CRC-DM was significantly higher than the non-diabetic colitis-CRC counterpart (Figure 6D). Combining the results from Figure 6C,D, pCRMP2 of CRC-DM mice was significantly higher than the non-diabetic colitis-CRC although their tCRMP2 levels were significantly reduced. As s-CRMP2 and pCRMP2 are required for cell division via mediating microtubule structure and appears to be a distinctive feature of highly proliferative cells [27], our findings suggest that, compared to colitis-CRC mice, increased inactive pCRMP2 in CRC-DM reflects advanced malignant potential probably due to pCRMP2-mediated cytoskeleton instability. Notably, considering the findings of histological characteristics, higher CRMP2/Ki-67 and pCRMP2 in the lesions of CRC-DM compared to the non-diabetic colitis-CRC mice further support the fact that diabetic hyperglycemia upregulates s-CRMP2/pCRMP2 and thus takes part in accelerating CRC progression.

## 4. Discussion

Our recent report provides molecular evidence supporting the link of pathogenesis leading to neurodegenerative and metabolic diseases by deciphering the involvement of the PARL-PINK1-Parkin system in adipogenesis [28]. Similar to this scenario, accumulating epidemiological evidence indicates that CRC and T2DM share common risk factors and etiology. However, substantial molecular studies elucidating the link between CRC and DM are scarce. We previously revealed that CRMP2 is significantly increased in adipose tissues of DIO mice, indicating that CRMP2 is associated with obesity and metabolic disorders [18]. In the present study, we aimed to investigate putative involvement of CRMP2 profile in serial disease progression from DIO, DM to CRC-DM by resorting to in vitro and in vivo study strategies. The present findings not only support the conclusion of our previous report [18] that f-CRMP2/s-CRMP2 and the corresponding phosphorylated status must be intricately modulated to ensure that the flexibility of the microtubule structure meets dynamic cellular needs, but also provide molecular evidence explaining common pathogenesis and/or disease progression between CRC and DM. From the perspective of CRMP2 expression patterns, CRMP2 is considered as a CRC biomarker [15], while s-CRMP2 is reported to control cell differentiation and proliferation, as well as correlate with poor prognosis of various cancers [12,16,29]. In support of the above reports, we previously demonstrated that s-CRMP2 is coupled with mitosis for the cells to acquire mitotic apparatus through mediating microtubule flexibility. The present study further reveals several intriguing findings supporting the participation of hyperglycemia and CRMP2 in CRC tumorigenesis and progression. First of all, while total CRMP2 is significantly increased in non-diabetic DIO and colitis-CRC mice, it is dramatically decreased in the corresponding diabetic DM and CRC-DM counterparts (Figure 6). The observation explains the significantly higher CRMP2 of CRC subjects than the patients with diabetic comorbidity (Table 2). Secondly, as colitis-CRC and CRC-DM mice served as the corresponding model for human CRC and CRC-DM subjects, the finding that hyperglycemia significantly downregulated CRMP2 (Figure 6C) not only further echoes lower CRMP2 detected in CRC-DM patients but also the association between glucose-mediated CRMP2 with CRC tumorigenesis [15,18]. By combining the above data, CRMP2 is likely to be upregulated in DIO probably due to chronic inflammation induced by insulin resistance but then prominently decreased after diabetic onset. Furthermore, the current observation that s-CRMP2 is the predominant form detected in CRC cell lines (Figure 3A and Figure 4A), animal (Figure 6B) and human CRC tissues (Figure 2A) supports our previous finding that s-CRMP2 performs as a dominant regulator over f-CRMP2 to direct cell proliferation and cytoskeleton dynamics, and thus has a pathological role in tumorigenesis [18]. In brief, the above data add evidence for the involvement of diabetic hyperglycemia-suppressed CRMP2 in CRC tumorigenesis and disease progression. Moreover, the findings support our previous inference that via manipulating CRMP2 profiles (including s-CRMP2/f-CRMP2 balance and pCRMP2 level) and thus the cytoskeleton dynamics by environmental glucose may determine proliferation and malignant characteristics of cancer cells.

In addition to the expression pattern, CRMP2 activity is tightly regulated by phosphorylation. Phosphorylated CRMP2 loses its tubulin-binding capacity and the ability to sustain microtubule assembly [30,31]. Therefore, increased pCRMP2 triggers tubulin depolymerization and microtubule destabilization that provides the cells higher morphological flexibility and mobility, and thus advanced metastatic capability. The finding of higher levels of inactive pCRMP2 in CRC-DM patients (Figure 2C) and mice (compared to colitis-CRC, Figure 6D) indicates that hyperglycemia downregulates CRMP2 and supports the association between glucose-mediated CRMP2 with CRC tumorigenesis [15]. Therefore, these results explain the underlying mechanisms of lower CRMP2 in CRC-DM patients and the correlation between CRC disease progression and diabetic onset in CRC-DM subjects (Table 2). Moreover, this study provides molecular data elucidating the contribution of diabetic hyperglycemia to disease progression and poor prognosis in various cancers via mediating cytoskeleton stability [12,17,29,32].

**Figure 7:**
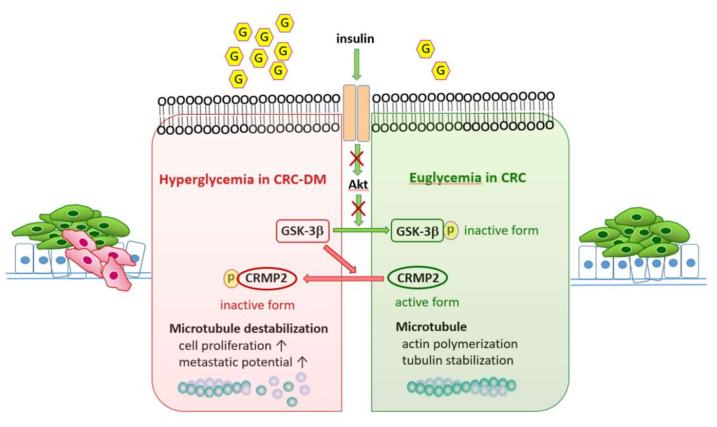
Working model of the contribution of hyperglycemia-suppressed CRMP2 activity in disease progression of CRC patients with diabetes comorbidity. In CRC cells (green-labeled cells) under physiological euglycemic microenvironment (illustrated as the right hand green shaded circumstance), insulin signaling is successfully received and transmitted intracellularly (as indicated by green arrows) to inactivate GSK-3β and mediate the subsequent CRMP2-associated actin polymerization and tubulin stabilization. On the contrary, in CRC-DM comorbidity with insulin resistance (represented as the left hand red shaded circumstance), hyperglycemia suppresses CRMP2 expression/activity and promotes actin de-polymerization due to impaired insulin signaling (as shown by red crosses) and augments the malignant characteristics of CRC cells with higher metastatic potential (red-labeled cells). Therefore, hyperglycemia mediates actin polymerization, cytoskeleton flexibility, and thus cell proliferation/metastasis by downregulating CRMP2 functional profile and thus contributes to CRC disease progression. In brief, diabetic hyperglycemia boosts the vicious effects to promote malignant characteristics via modulating CRMP2, and thus leads to advanced tumor stage. G in hexagon: glucose; p in circle, phosphate group.

The observations derived from in vitro, animal, and clinical strategies strongly support the participation and implication of glucose and CRMP2 in metabolic efficacy, tumorigenesis, and disease progression via regulating microtubule remodeling. In this context, it is reasonable to infer that disturbed homeostasis of CRMP2-associated cytoskeleton dynamics by environmental glucose participates in pathogenesis leading to metabolic [18] and cancerous consequences. In brief, diabetic hyperglycemia further boosts the vicious effects to promote malignant characteristics via modulating CRMP2, and thus leads to advanced tumor stage. Thus, as depicted in the working model (Figure 7), CRC cells with suppressed CRMP2 expression and activity in CRC-DM patients tend to have higher basement membrane-penetration, migration, and metastatic potential due to cytoskeleton instability and loose cell–cell adhesion, which eventually result in tumor progression and advanced staging. Our results not only verify that CRMP2 is a CRC tumor marker [15] but provide molecular evidence supporting CRC and diabetes comorbidity, as well as the involvement of glucose in CRC disease progression via mediating the CRMP2-regulated cytoskeleton.

Discrepancies regarding CRMP2 expression in cancers are reported. While CRMP2 levels are not correlated with clinical outcomes of cancer patients [15,27], the significantly increased nuclei-localized pCRMP2 in advanced tumors is suggested to contribute to cancer progression and thus is significantly associated with lower survival rate [27,32]. As the majority of signaling molecules exert their functions through mediating CRMP2 phosphorylation and thus its function, these results indicated that pCRMP2 levels and nuclear localization are more important than total CRMP2 levels. Further study is required to elucidate the exact functions of CRMP2, particularly with regard to phosphorylation and nuclear localization in CRC.

On the other hand, we hypothesized that the anti-inflammatory cytokine interleukin-4 (IL-4) was able to attenuate DIO-induced CRMP2 expression based on its multiple function to promote energy catabolism and ameliorate diabetic progression [18,25,26,33,34,35,36]. Therefore, CRMP2 expression profile was also analyzed in DIO mice receiving IL-4 administration to test the involvement of CRMP2 in DIO. As anticipated, total CRMP2, f-CRMP2, and s-CRMP2 were all significantly downregulated in DIO mice by IL-4 administration (Supplementary Figure S1). The results imply that IL-4 counteracts the obesity-derived metabolic abnormalities, including DIO-induced CRMP2 expression. The attenuation of DIO-induced s-CRMP2 by IL-4 further verifies our inference that CRMP2 activity is elevated during the process of developing obesity. Accordingly, CRMP2 levels and activity are crucial and must be finely tuned for maintaining dynamic homeostasis of cytoskeleton structure and flexibility of mechanical strength in response to cellular needs.

There are limitations in the present study. Firstly, CRMP2 expression is lower accompanied by aging in animal models [37]. The exacerbated neural degenerative symptoms in diabetic patients with increasing age is suggested to be correlated with decreased CRMP2 [38]. Therefore, the possibility of the age-coupled CRMP2 level cannot be excluded in this study owing to the significant age difference between CRC and CRC-DM subjects. Secondly, the exact CRMP2 form which is decreased in tumor cells and thus contributed to disease progression in CRC-DM patients awaits further investigation since the antibodies used in the IHC staining were not able to differentiate f-CRMP2 from s-CRMP2. Moreover, we could only provide CRMP2 expression profiles from a very limited number of subjects (Figure 2; 5 CRC and 1 CRC-DM) due to the availability of CRC fresh tissues from clinical settings. Nevertheless, the parallel results from human and animal models support the involvement and implication of glucose-mediated CRMP2 in CRC tumorigenesis and progression. Fourthly, the effects of CRMP2 KD in transient SW620 transfectants and the short-term (72 h) of glucose treatment on CRC cells were investigated, which cannot faithfully reflect long-term influences of low CRMP2 expression and hyperglycemia to CRC cells. In addition, CRMP2 overexpression endowed HCT15 cells with unexpectedly tight adherence and thus the migration ability was not successfully analyzed. 

## 5. Conclusions

To the best of our knowledge, this is the first report providing novel clues to substantiate and elucidate the link between CRC and T2DM, as well as characterizing the roles of CRMP2 in CRC-DM. As depicted in Figure 7, in addition to causing multiple diabetic complications, hyperglycemia in diabetic patients mediates cytoskeleton remodeling and thus enhances cell proliferation/metastasis via promoting actin de-polymerization by downregulating CRMP2 functional profile and thus contributes to CRC disease progression. In summary, persistent downregulation of CRMP2 by hyperglycemia promotes CRC progression in patients with diabetic comorbidity. As novel roles and physiological functions of CRMP2 are characterized, the impact of CRMP2 dysregulation and the corresponding clinical application deserve further investigation. Furthermore, as diabetic prevalence is exponentially increasing globally and impaired metabolic homeostasis is identified in numerous cancer types, these altered metabolic adaptions are promising targets for the development of anti-diabetic and cancer strategies.

## Figures and Tables

**Table 1:** Characteristics of CRC and CRC-DM patients in this study.

Subject Characteristics	CRC (61)	CRC-DM (46)	*p*
Gender			
Male	41 (67.2)	29 (63.0)	0.653 ^#^
Female	20 (32.8)	17 (37.0)	
Hypertension			
Yes	26 (42.6)	28 (60.9)	0.062 ^#^
No	35 (57.4)	18 (39.1)	
Stage			
Ⅰ	11 (19.0)	6 (14.6)	0.487 ^#^
Ⅱ	28 (48.3)	16 (39.0)	
Ⅲ	16 (27.6)	14 (34.1)	
Ⅳ	3 (5.2)	5 (12.2)	
CRMP2 expression ^‡^			
Low	17 (27.9)	36 (78.3)	<0.0001 ^#^
High	44 (72.1)	10 (21.7)	
Age	68.2 ± 13.6	73.5 ± 11.5	0.012 ^¶^
Body mass index	25.1 ± 3.9	25.5 ± 4.7	0.607 ^¶^
Total cholesterol (125–240 mg/dL) ^†^	170.2 ± 38.8	171.4 ± 36.0	0.867 ^¶^
Triglycerides (20–200 mg/dL) ^†^	91.1 ± 45.8	127.3 ± 76.0	0.007 ^¶^

^†^ Data were presented as mean ± standard deviation, numbers in parenthesis indicated the normal reference range of each biochemical test. ^‡^ CRMP2 expression of each study subject was categorized by combining the 2 scores of the percentages of cells showing cytosolic positive staining results and staining strength as described in Section 2. CRMP2 expression was recorded as low expression (L) with scores 0^+^ and 1^+^, and as high expression (H) with scores >2^+^. ^#^ Fisher’s Exact Test, Two-sample T-test.

**Table 2:** CRMP2 expression in CRC and CRC-DM study subjects.

CRMP2 Expression ^#^	CRC/n (%) ^‡^	CRC-DM/n (%) ^‡^	*p* *	Odds Ratio (95%CI)
0^+^	4 (6.6%)	12 (26.1%)	-	-
1^+^	13 (21.3%)	24 (52.2%)	0.470	0.615 (0.165~2.298)
2^+^	25 (41.0%)	8 (17.4%)	0.002	0.107 (0.027~0.426)
3^+^	19 (31.1%)	2 (4.3%)	<0.0001	0.035 (0.006~0.222)

^#^ CRMP2 expression of each study subject was documented and categorized by the 2 scores of the percentages of cells showing cytosolic positive staining results and staining strength as described in Section 2. The 0~2 recorded as 0^+^ (weakest expression), 3~4 as 1^+^, 5~6 as 2^+^, and 7~8 as 3^+^ (strongest expression). ^‡^ Data were presented as the number and percentage of patients in the study group. * Logistic analysis using CRMP2 expression 0^+^ as the reference group.

## Data Availability

All data generated or analyzed during this study are included in this published article and the supplementary information files.

## Supplementary Materials

The following are available online at https://www.mdpi.com/article/10.3390/cells11040727/s1, Figure S1: Regulation of CRMP2 in adipose tissue under diabetic and insulin resistance status.
