# Supplementary material for: Characterization of Collapsin Response Mediator Protein 2 in Colorectal Cancer Progression in Subjects with Diabetic Comorbidity
Source: Cells. 2022 Feb 18;11(4):727. doi: 10.3390/cells11040727 (PMC8869905; doi:10.3390/cells11040727)
Supplement: Supplementary file 1 [file cells-11-00727-s001.zip › cells-1574332-supplementary.pdf]

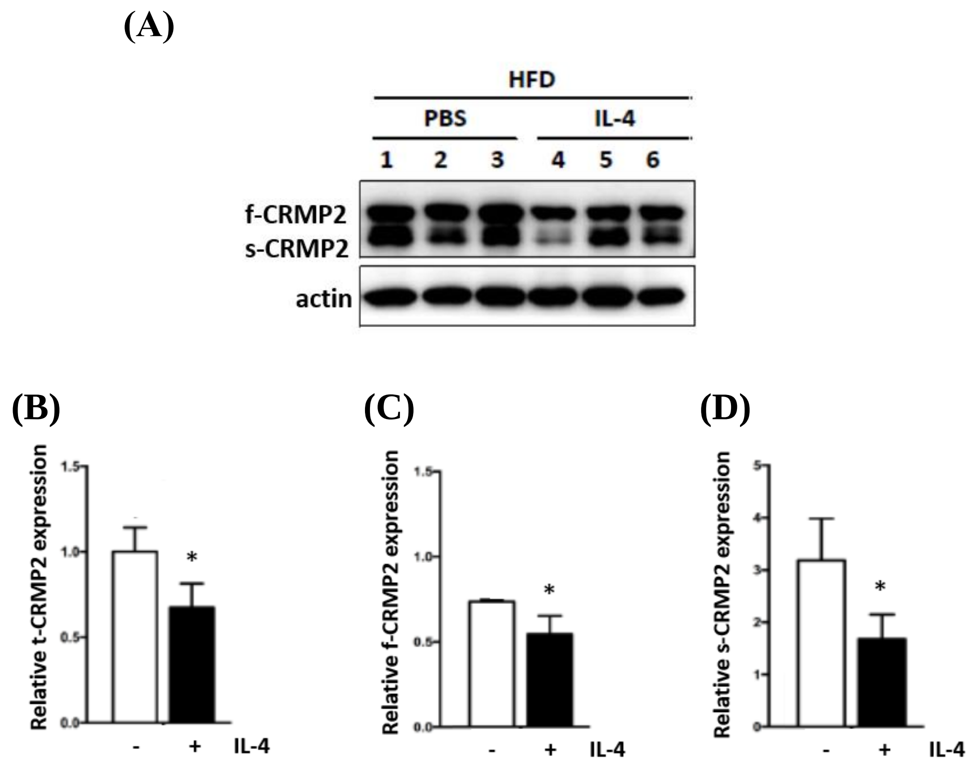

Figure S1. Regulation of CRMP2 in adipose tissue under diabetic and insulin resistance status.

(A) Epididymal adipose tissues were obtained from mice fed with chow diet or high fat diet (HFD)-induced insulin resistance with IL-4 administration. Alternations of CRMP2 were analyzed by Western blots. (B-D) The quantitative results from (A) were presented as the mean ± SEM (n=3), $^{*}p<0.05$, $^{***}p<0.005$. ($^{\#}$The HFD mice with PBS treatment were the same mice group published in ref. 18.)
